# Efficient elimination of RNA mycoviruses in *aspergillus* species using RdRp-inhibitors ribavirin and 2’-C-methylribonucleoside derivatives

**DOI:** 10.3389/fmicb.2022.1024933

**Published:** 2022-10-06

**Authors:** Ayano Ikeda, Yuto Chiba, Misa Kuroki, Syun-ichi Urayama, Daisuke Hagiwara

**Affiliations:** ^1^Laboratory of Fungal Interaction and Molecular Biology (Donated by IFO), Department of Life and Environmental Sciences, University of Tsukuba, Tsukuba, Ibaraki, Japan; ^2^Microbiology Research Center for Sustainability (MiCS), University of Tsukuba, Tsukuba, Ibaraki, Japan

**Keywords:** RNA virus, aspergillus, mycovirus, nucleoside analog, antiviral drug

## Abstract

RNA viruses in fungi (mycoviruses) are model systems for understanding the relationships between eukaryotic microorganisms and RNA viruses. To reveal the effects of mycoviruses on host fungi, it is essential to compare the phenotypes between isogenic fungal isolates with or without RNA virus infection. Since active entry machinery for RNA mycoviruses has never been identified, introducing mycoviruses to fungi is a difficult and time-consuming process. Therefore, most studies have tried to generate virus-free isolates from infected strains by eliminating the mycovirus. However, methods of elimination have not been evaluated in a quantitative and comparative manner. In this study, we established a method to remove mycoviruses from host cells using the antiviral drugs ribavirin, 2′-C-methylcytidine (2CMC), 2′-C-methyladenosine (2CMA), and 7d2CMA, and compared the efficiency of removal in virus-infected strains of *Aspergillus fumigatus*. The results indicated that treatment with the drugs removed RNA viruses of diverse proportions in the families *Chrysoviridae, Mitoviridae, Partitiviridae, Polymycoviridae,* and an unclassified RNA virus group. Viruses belonging to *Narnaviridae* were hardly eliminated by these antiviral treatments when they were the sole infectious agents. We found that 2CMC showed activity against a wider range of RNA mycoviruses compared to ribavirin, 2CMA, and 7d2CMA, although 7d2CMA also efficiently removed dsRNA viruses from the families *Chrysoviridae*, *Partitiviridae*, and *Polymycoviridae*. These results indicated that removal of mycoviruses depends on the specific viral species and antiviral drug. This is the first report demonstrating a preferential antiviral effect against mycoviruses, which will enhance research on microbial RNA viruses and support their elimination from economically important fungi such as edible mushrooms.

## Introduction

RNA viruses have long been identified and isolated from humans and economically important and diseased organisms such as livestock and crops (Beijerinck, 1898; Loeffler and Frosch, 1898; Reed and Carroll, 1901). However, in the last decade, thousands of new RNA viruses have been identified from wild organisms, and thus researchers have realized that RNA viruses are ubiquitous (Roossinck et al., 2010; Shi et al., 2016, 2018; Edgar et al., 2022; Hirai et al., 2022; Neri et al., 2022; Zayed et al., 2022). In particular, microbial RNA viruses are thought to be a major reservoir of the RNA virosphere on Earth. New RNA viruses have constantly been identified from (meta-)transcriptomic data in various environments (Urayama et al., 2018; Starr et al., 2019; Sutela et al., 2019; Hillary et al., 2022), which expands our knowledge of the RNA virosphere. However, difficulty in isolating diverse host microorganisms with RNA viruses hampers the characterization of those viruses from a biological and ecological viewpoint (Urayama et al., 2022).

Fungal RNA viruses have a persistent-type lifecycle in which they spread mainly vertically, without infection of other cells and release from the host. Therefore, most of them do not show severe pathogenicity to their host fungi (Nuss, 2005; Son et al., 2015). Due to the lack of knowledge on the functions of RNA viruses, their importance to the biosphere containing their fungal hosts and other organisms remains unclear. To reveal the biological and ecological significance of these RNA viruses, we need to compare phenotypes between fungal isolates harboring RNA viruses and isogenic fungal isolates without them (Cao et al., 2019; Takahashi-Nakaguchi et al., 2020). Importantly, RNA mycoviruses have no active entry machinery (Nuss, 2005). Therefore, generating virus-infected fungal strains by artificial introduction of the mycoviruses is difficult. Because of this, most studies have employed virus removal from RNA virus-harboring isolates to prepare sets of virus-infected and virus-free strains (Marquez et al., 2007; Kotta-Loizou and Coutts, 2017; Okada et al., 2018; Sato et al., 2022). For example, Takahashi-Nakaguchi and co-workers isolated AfuCV-free strain from *A. fumigatus* IFM 41362, and compared the phenotypes of these two strains to reveal the effect of AfuCV (Takahashi-Nakaguchi et al., 2019).

In general, virus removal is thought to be an incidental event during fungal growth under laboratory and environmental conditions (Sato et al., 2022). It is empirically recognized that culture conditions, virus type, and host strain are the major determinants of virus elimination efficiency. Several experiments using heat stress, hyphal tip cultures, protein synthesis inhibitors (cycloheximide), antibiotics, or antiviral compounds have been reported to generate virus-free strains (Kwon et al., 2012; Kotta-Loizou and Coutts, 2017; Applen Clancey et al., 2020). However, these experiments have been employed in different studies with different experimental conditions using different host fungal species and virus species. Therefore, the effects of these treatments have not been generalized. Furthermore, efficient methods applicable to many fungal RNA viruses have never been developed.

Ribavirin (1-β-d-ribofuranosyl-1,2,4-triazole-3-carboxamide), a guanine nucleoside analog, is a broad-spectrum inhibitor of RNA virus replication (Leyssen et al., 2008). The mode of action for ribavirin remains a matter of debate, but it is used as a hepatitis C virus (HCV) therapeutic drug. To eliminate RNA viruses from fungal isolates, ribavirin has been used in several reports. Herrero & Zabalgogeazcoa reported the successful removal of a dsRNA virus (*Totiviridae*) from *Tolypocladium cylindrosporum* with 100% efficiency (Herrero and Zabalgogeazcoa, 2011). However, another unknown RNA virus infecting the same isolate was not eliminated by the treatment with ribavirin. Niu et al. also reported the successful removal of *Polymycoviridae* (dsRNA) and *Narnaviridae* (ssRNA) using a combinational approach of protoplast regeneration and ribavirin treatment (Niu et al., 2018). However, ribavirin treatment has failed to remove viruses in *Fusariviridae* (proposed family, ssRNA), *Mitoviridae* (ssRNA), and *Hypoviridae* (ssRNA; Park et al., 2006; Liu et al., 2015; Velasco et al., 2018). These studies were performed under different conditions and used different fungal species, and the number of trials and elimination efficiency were not shown in most of these studies. Also, in some cases, other viruses in the same cells and other treatments such as protoplast regeneration could affect the results. Therefore, the applicability of ribavirin to fungal RNA virus research is still under consideration.

2′-C-methylcytidine (2CMC) is a member of the 2’-C-methylribonucleoside class of compounds. This class includes 2′-C-methyladenosine (2CMA) and the modified form 7-deaza-2′-C-methyladenosine (7d2CMA), which has previously been shown to inhibit RNA-dependent RNA polymerase (RdRp), the only universal gene among RNA viruses, of HCV (Carroll et al., 2003; Olsen et al., 2004). Kuhlmann et al. reported the first evidence of eliminating persistent-type dsRNA viruses (*Totiviridae*) in the protist *Leishmania* by 2CMA and 7d2CMA (Kuhlmann et al., 2017). In that study, 2CMA and 7d2CMA reduced viral dsRNA, but 2CMC did not. In contrast, Narayanasamy et al. reported the effect of these compounds on totiviruses infecting the protist *Trichomonas* (Narayanasamy et al., 2022), and 2CMC treatment for 48 h removed the viruses with 100% efficiency (10/10 clones), but 2CMA did not. These reports suggest that some 2’-C-methylribonucleoside class compounds can enhance the removal of *Totiviridae* viruses infecting protists. However, the usability of these compounds in fungal RNA virus research has never been reported.

According to the reports described above, treatment with antiviral drugs is a promising conventional method for removing mycoviruses from fungal hosts. However, there has been no quantitative evaluation of the efficiency of RNA mycovirus elimination. Therefore, in this study, we evaluated the impact of the antiviral compounds ribavirin and 2’-C-methylribonucleosides for obtaining virus-free fungal isolates. To achieve this, we used seven RNA viruses classified into different families infecting the single species *Aspergillus fumigatus* (Chiba et al., 2021). Our data present the first overview of antiviral drugs for several mycoviruses and provide a method for efficient RNA virus elimination.

## Materials and methods

### Fungal strain and culture conditions

Fungal strains and RNA viruses used in this study are listed in Table 1. The strains with an IFM number were provided by the Medical Mycology Research Center, Chiba University through the National Bio-Resource Project, Japan,^1^ and *Penicillium chrysogenum* JCM2056 was purchased from RIKEN JCM.^2^

**Table 1:** Viruses and hosts used in this study.

Host name	Host accession	Virus name	Abbreviation	Virus accession	Genome type	Family
*Aspergillus fumigatus*	IFM63439	Aspergillus fumigatus RNA virus 1	AfuRV1	txid2747487	positive-ssRNA	unclassified
*Aspergillus fumigatus*	IFM64916	Aspergillus fumigatus botourmiavirus 1	AfuBOV1	txid2747486	positive-ssRNA	*Botourmiaviridae*
*Aspergillus fumigatus*	IFM62355	Aspergillus fumigatus mitovirus 1	AfuMV1	txid2250451	positive-ssRNA	*Mitoviridae*
*Aspergillus fumigatus*	IFM63431	Aspergillus fumigatus narnavirus 2	AfuNV2	txid2250450	positive-ssRNA	*Narnaviridae*
*Aspergillus fumigatus*	IFM62632	Aspergillus fumigatus polymycovirus 1	AfuPmV-1	txid2250469	dsRNA	*Polymycoviridae*
		Aspergillus fumigatus negative-stranded RNA virus 1	AfuNSRV1	txid2749925	negative-ssRNA	*Betamycobunyaviridae* (suggested)
*Aspergillus fumigatus*	IFM63147	Aspergillus fumigatus chrysovirus	AfuCV	txid607716	dsRNA	*Chrysoviridae*
		Aspergillus fumigatus narnavirus 2	AfuNV2	txid2250450	positive-ssRNA	*Narnaviridae*
		Aspergillus fumigatus botourmiavirus 1	AfuBOV1	txid2747486	positive-ssRNA	*Botourmiaviridae*
*Penicillium chrysogenum*	JCM2056	Penicillium chrysogenum virus	PcV	txid158372	dsRNA	*Chrysoviridae*
*Aspergillus lentulus*	IFM 62627	Aspergillus lentulus partitivirus 1	AlePV1	txid2747488	dsRNA	*Partitiviridae*
*Aspergillus fumigatus*	IFM61469	Aspergillus fumigatus partitivirus 1	AfuPV1	txid1027415	dsRNA	*Partitiviridae*
*Aspergillus fumigatus*	Af293	Aspergillus fumigatus polymycovirus 1	AfuPmV-1	txid2250469	dsRNA	*Polymycoviridae*

These strains were cultured in potato dextrose agar (PDA) plate media for 5 days at 37°C. Conidia were harvested with 0.05% Tween 20 and then passed through a 40-μm nylon mesh. For treatment with antiviral compounds, the conidia were dropped on the center of PDA containing antiviral compounds and incubated for 5 days at 37°C. The final concentrations of each compound were as follows: ribavirin (Cayman Chemical, Ann Arbor, MI, United States), 0.1 mg/ml; 7-Deaza-2’-C-Methyladenosine (7d2CMA; Carbosynth Ltd., United Kingdom), 0.2 mg/ml; 2’-C-Methyladenosine (2CMA; Carbosynth), 0.2 mg/ml; 2’-C-Methylcytidine (2CMC; Carbosynth), 0.2 mg/ml. DMSO was used as the solvent for these drugs, and DMSO without compounds (final concentration: 10 μl/ml for ribavirin, 2 μl/ml for other drugs) was used for the negative-control PDA. In these conditions, clear fungal growth inhibition was not observed with any compound. For liquid cultures, the strains were cultured in potato dextrose broth (PDB) with reciprocal shaking (120 rpm) for up to 5 days at 37°C. *P. chrysogenum* was also cultured using PDA or PDB at 25°C.

### Preparation of drug-treated spores and single colony formation

Drug-treated spores were obtained from five mycelial plugs. Conidia were harvested with 0.05% Tween 20 and then passed through a 40-μm nylon mesh. The concentration of spores was adjusted to 50 spores/ml. To obtain single colonies, 500 μl of spore solution was spread on yeast extract peptone dextrose agar [YPDA: Bacto Yeast Extract 1%, Bacto Peptone (BD) 2%, D-(+)-glucose (Nacalai tesque) 2%, Agar 1.5%] media and incubated for 1 day at 37°C (25°C for *P. chrysogenum*).

### Total nucleic acid extraction

To obtain template total nucleic acids for One-step RT-PCR, fungal cells (0.1–0.2 g) were disrupted using FastPrep 24 (MP Biomedicals, Santa Ana, CA, United States) in 2× STE (0.2 M Tris–HCl, 0.2 M NaCl, and 2 mM EDTA, pH 6.8) containing 0.1% (v/v) β-mercaptoethanol. Total nucleic acids were manually extracted with sodium dodecyl sulfate-phenol.

### One-step RT-PCR and sanger sequencing

For the detection of RNA viruses in fungal colonies, direct one-step RT-PCR was applied as described previously (Urayama et al., 2014). The colonies were picked by a toothpick, and the toothpick was dipped in 10 μl one-step RT-PCR reaction mix [Super-Script III One-Step RT-PCR System with Platinum Taq (Invitrogen, Carlsbad, CA, United States)] in a PCR tube and twisted three times. The primers used in this study are listed in Supplementary Table S1. Reverse transcription at 55°C for 20 min and 60°C for 10 min was followed by 2 min at 94°C to activate DNA polymerase; for PCR, 40 cycles of denaturation at 94°C for 15 s, annealing at 55°C for 30 s, and extension at 68°C for 1 min was followed by a final extension for 5 min at 68°C. The products were subjected to agarose gel electrophoresis and visualized with GelRed staining (Biotium, Inc., Fremont, CA, United States) under UV light. Some representative bands were subject to Sanger sequencing. To detect viral RNA among total nucleic acids, we also used a similar system.

## Results

### Establishment of a quantitative evaluation system for antiviral drug effects

**Figure 1:**
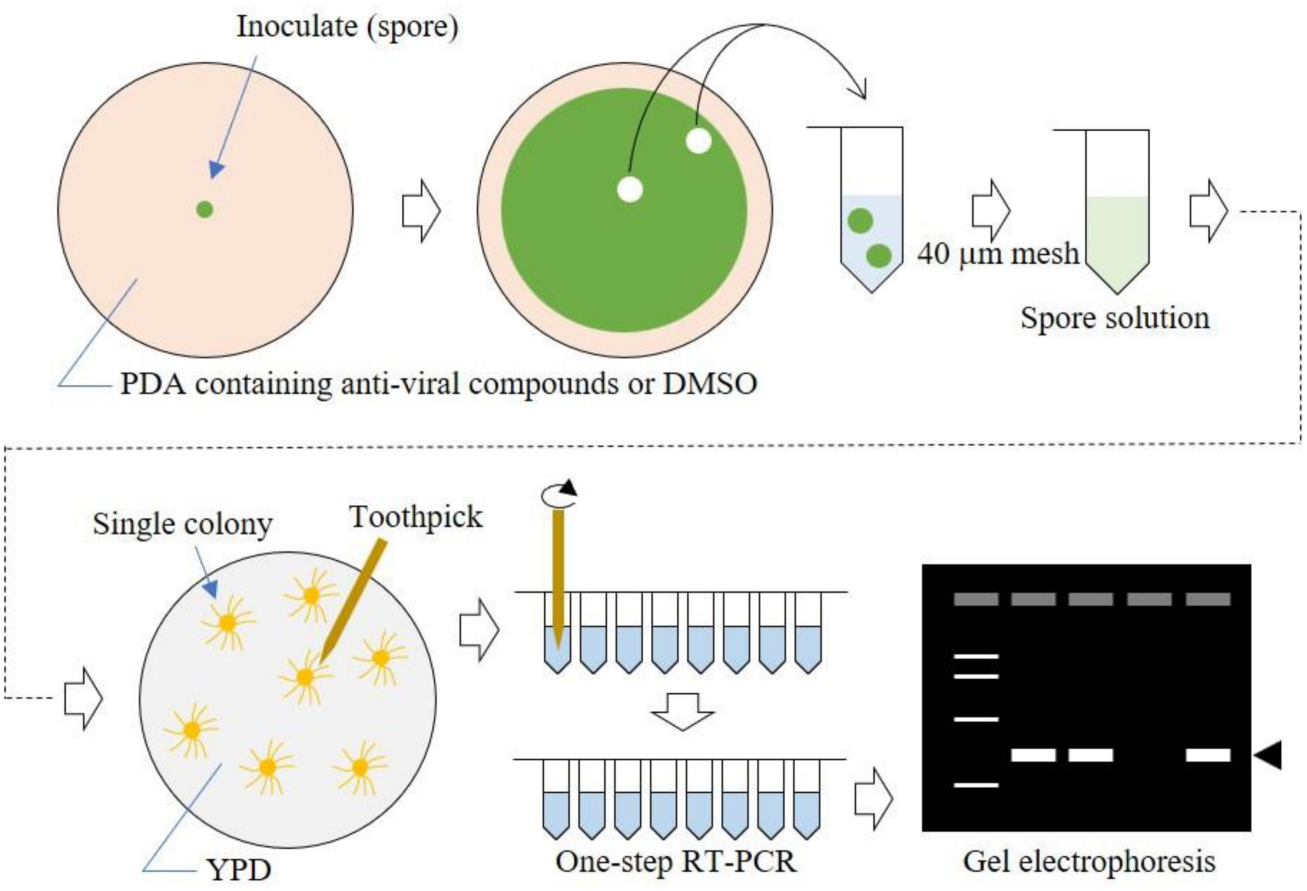
Schematic workflow of our method. Fungi were cultured on nucleoside analog-containing plate media, then spores were spread on YPD plate media to form single colonies. Using toothpicks as described previously (Urayama et al., 2014), viral RNA was amplified by one-step RT-PCR. PCR products were detected by electrophoresis.

Fungi are known for their multicellular and elongated cell structures. These features make it difficult to quantitatively evaluate virus elimination efficiency in each cell. Here, we took advantage of the persistent-type lifecycle of mycoviruses, namely vertical transmission of viruses *via* conidia, to judge the efficacy of antiviral drugs in virus elimination. The scheme of antiviral drug treatment is shown in Figure 1. First, a virus-infected fungal strain is inoculated on the center of a medium plate containing an appropriate concentration of the antiviral drug. After colony growth for up to 5 days, agar plugs are cut from the central and outside regions of the plate. A conidia suspension is generated from the agar plugs, and single colonies are obtained by spreading the conidia on YPDA plate media. Viruses in a single colony are detected by one-step RT-PCR. The rationale is that the proportion of virus-free colonies reflects the virus-free conidia in the suspension. Consequently, the antiviral effects during colony growth and conidiation can be evaluated in a quantitative manner.

### Comparative study of the effects of ribavirin on multiple mycoviruses

**Figure 2:**
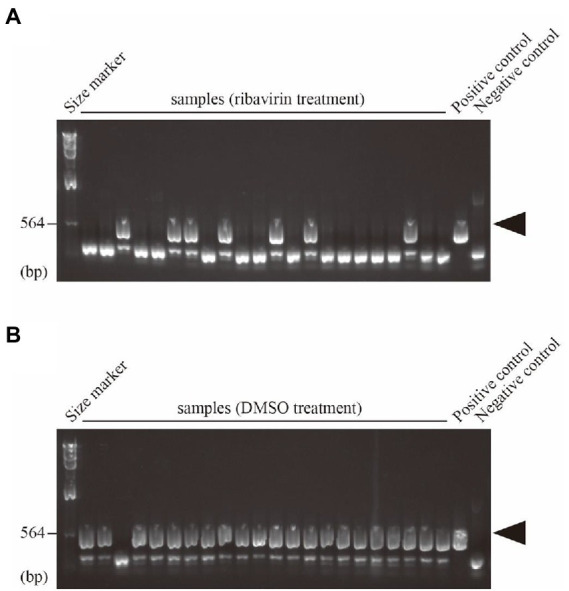
Direct detection of RNA1 of AfuRV1 in single conidial isolates treated by **(A)**, ribavirin or **(B)**, DMSO. Amplified fragments by one-step colony RT-PCR were electrophoresed on a 1% agarose gel with Lambda HindIII fragments as size markers. The primers are shown in Supplementary Table S1. Positions of the RT-PCR amplicons of AfuRV1 are shown by arrowheads.

To evaluate the effect of drugs, it is essential to compare the efficiency of virus removal among multiple viral species in an identical fungal species. We previously surveyed mycoviruses in more than a hundred strains of *A. fumigatus* and identified six host strains with seven different viruses (Chiba et al., 2021). First, to test the above evaluation system, one of the virus-infected strains, *A. fumigatus* IFM 63439 harboring Aspergillus fumigatus ssRNA virus 1 (AfuRV1, unclassified family), was treated with ribavirin. When 22 colonies from ribavirin-treated spores were checked, 15 colonies were determined to be virus-free (Figure 2A), whereas one virus-free colony was isolated without ribavirin treatment as a control (Figure 2B).

**Figure 3:**
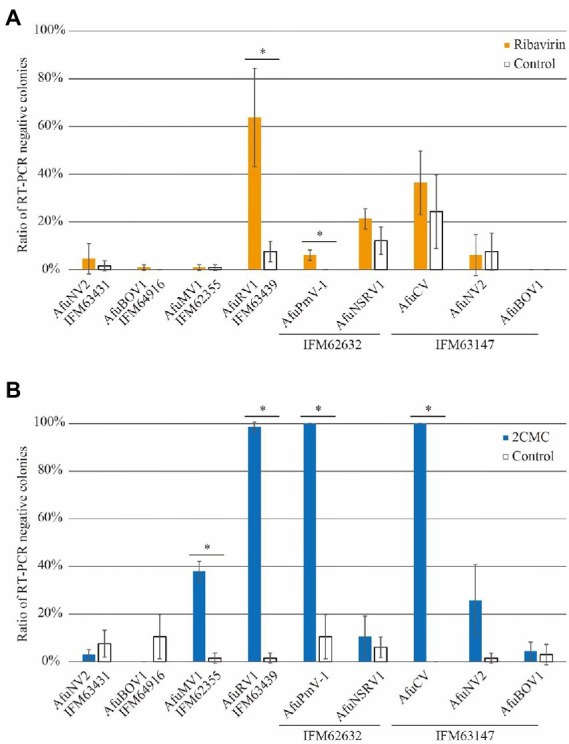
Ratios of virus-free colonies after treatment by **(A)**, ribavirin or **(B)**, 2CMC. Error bars represent SD and were calculated from three biological replicates. Asterisks indicate significant differences (*p* < 0.05; Student’s *t* test).

To expand our study, we treated other fungal strains with ribavirin, and virus-free colonies were detected using a set of specific primers for each viral sequence and calculated from three biological replicates. Among seven viruses, AfuRV1 was removed at the highest rate of over 60%, whereas the removal rate was less than 10% without ribavirin (Figure 3A). The fungal strain with AfuPmV-1 showed no spontaneous virus removal in the DMSO control, but the treatment enhanced the elimination rate (Figure 3A). Notably, some viruses showed spontaneous removal in the DMSO control. Given that control treatment with DMSO might affect the maintenance of viruses, the effects of ribavirin treatment on five other viruses (AfuNV2, AfuBOV1, AfuMV1, AfuNSRV1, and AfuCV) were tested and were not significant (Figure 3A).

To firmly check viral elimination, RNA was extracted from some strains that showed no PCR products by colony-direct one-step RT-PCR, and one-step RT-PCR using total nucleic acids was performed. In this evaluation, total nucleic acids extracted from 38 strains were obtained from the first trial with ribavirin treatment (left column of ribavirin treatment in Supplementary Table S2). As a result, RT-PCR products were not detected from 32 strains, but 6 strains were positive for viruses. The six strains harbored AfuNV2, AfuBOV1, or AfuMV1. This data suggested that our colony-direct one-step RT-PCR data included a low rate of false-negatives.

### Application of 2CMC for mycovirus removal

Ribavirin has been used for mycovirus removal in some studies; however, the other antiviral drugs had yet to be investigated. Thus, we next evaluated 2CMC for its ability to remove mycoviruses in infected in *A. fumigatus* strains. As in the case of ribavirin, AfuRV1 was preferentially removed at the highest rate (~97%; Figure 3B). The spontaneous virus elimination rate was low, which resulted in a significant increase of the elimination rate by 2CMC treatment. In addition, 2CMC also enhanced the appearance of AfuPmV-1-free and AfuCV-free fungal strains. Furthermore, more than 20% of the fungal strains became free of AfuMV1 and AfuNV2 (in IFM 63147). These results together suggest that 2CMC is effective in removing several species of mycovirus.

### Evaluation of other 2’-C-methylribonucleoside class compounds

**Figure 4:**
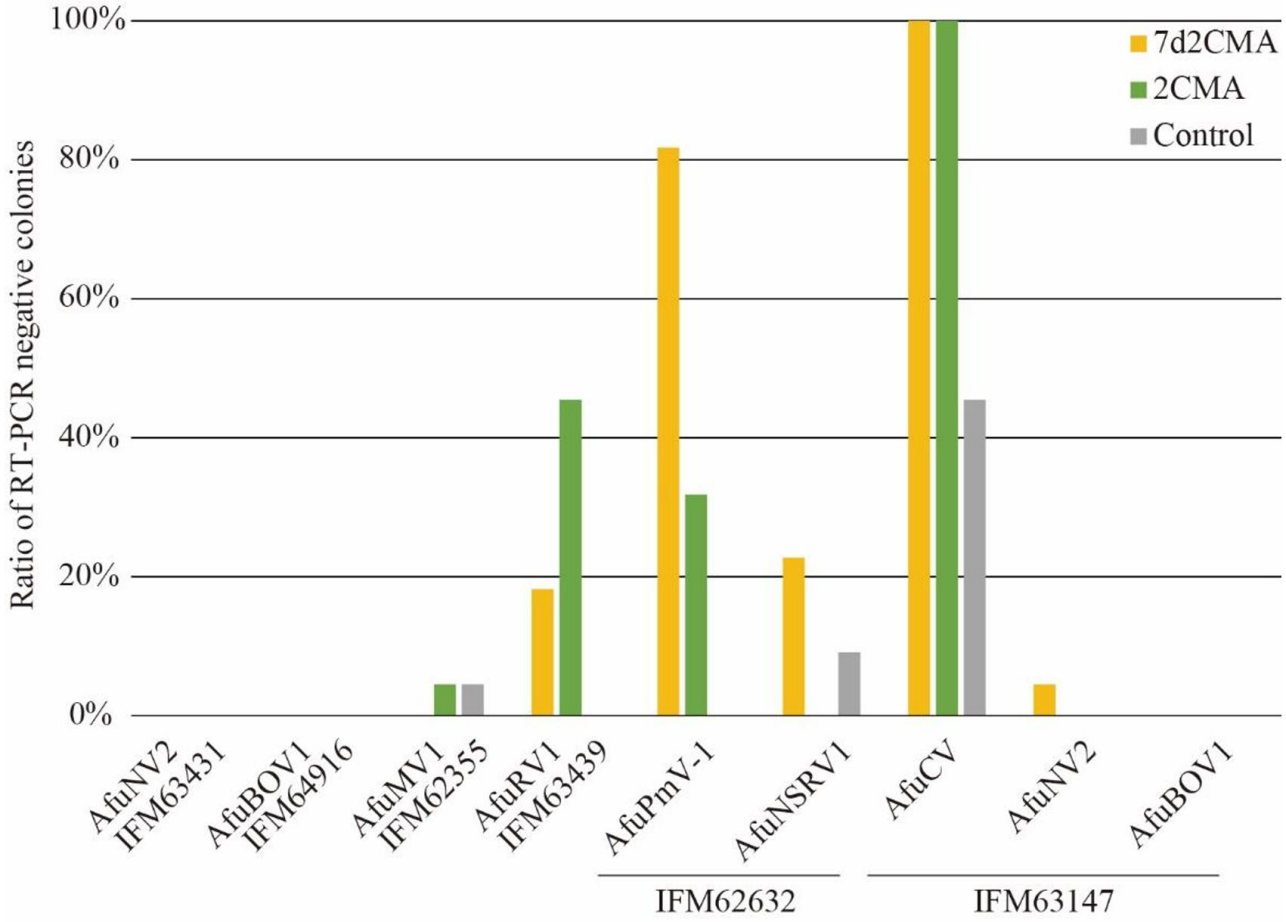
Ratios of virus-free colonies in *A. fumigatus* species after treatment by 7d2CMA or 2CMA.

Ribavirin and 2CMC are typical nucleoside analogues that inhibit viral RNA replication, probably by accessing the nucleoside binding pocket of the RdRp protein. Given that the mode of action is associated with mycovirus elimination, other nucleoside analogues could affect mycoviruses. To test this, we chose 2CMA, an adenine analogue, and 7d2CMA, which is a chemically-modified 2CMA, to treat virus-infected *A. fumigatus*. When treated with 2CMA, more than 30% of virus-free isolates were obtained for AfuRV1 and AfuPmV-1, whereas AfuCV was removed in all strains. Similarly, 7d2CMA treatment resulted in more than 80% of virus-free isolates for AfuPmV-1, whereas AfuCV was removed in all strains (Figure 4).

### Efficient removal of dsRNA mycoviruses by 2’-C-methylribonucleoside class compounds

**Figure 5:**
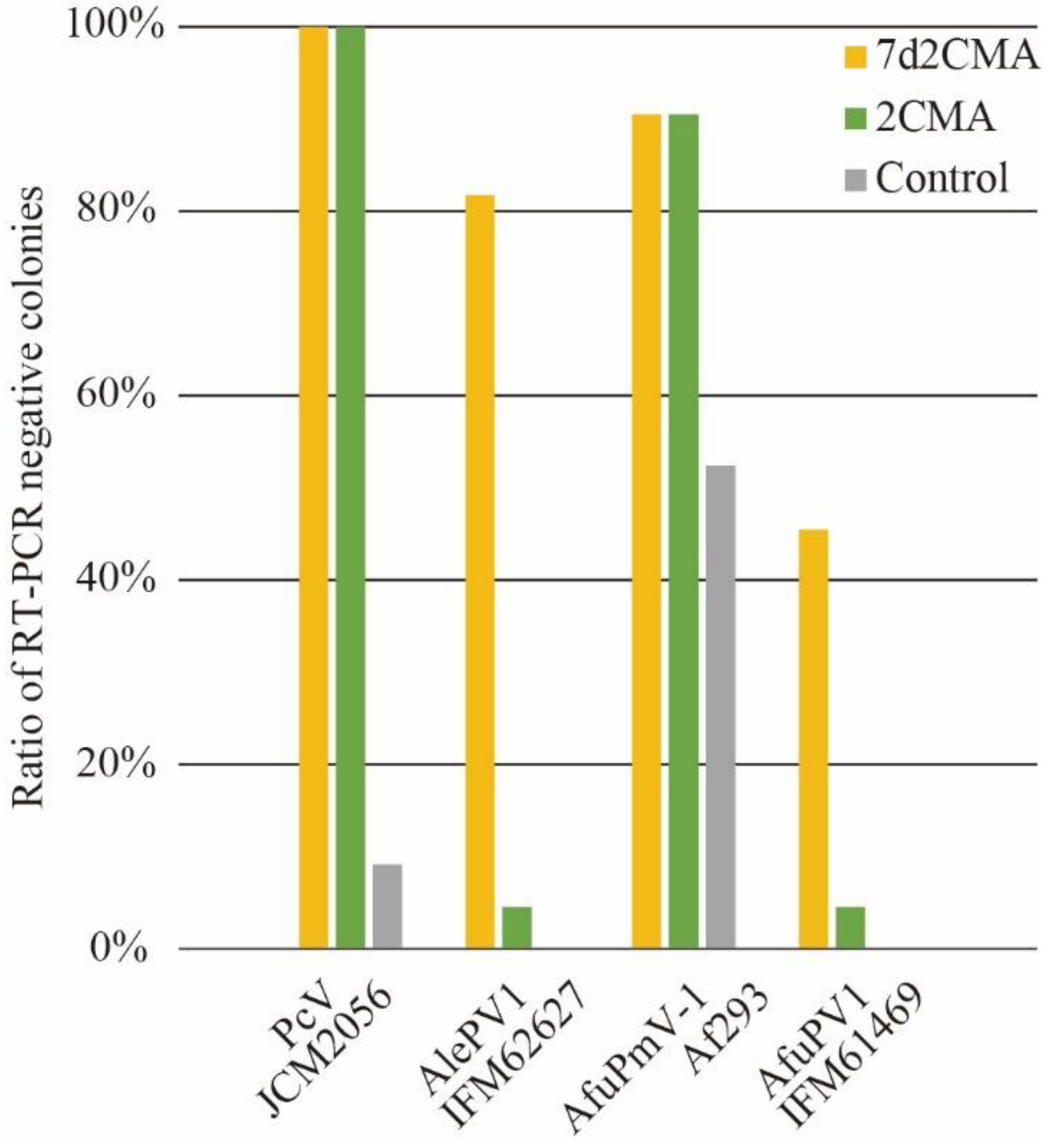
Ratios of dsRNA virus-free colonies after treatment by 7d2CMA or 2CMA.

Among the virus species tested here, the viruses with dsRNA genomes were AfuPmV-1 and AfuCV. Notably, both AfuPmV-1 and AfuCV tended to be efficiently removed by treatment with 2’-C-methylribonucleoside class compounds (Figure 5; Supplementary Table S3). To investigate whether these nucleoside analogue drugs are highly efficacious against dsRNA viruses, we also performed assays using 2CMA and 7d2CMA against an additional four dsRNA viruses: a chrysovirus, polymycovirus, and two partitiviruses. As reported previously, the *A. fumigatus* Af293 strain is infected by a polymycovirus (Takahashi-Nakaguchi et al., 2020), so it was used to test treatment with the drugs. As a result, 90% of the colonies were determined to be virus-free (Figure 5). Similarly, a *P. chrysogenum* strain infected with chrysovirus was treated with 2CMA and 7d2CMA. This strain was also efficiently cured of its virus. *A. fumigatus* IFM 61469 (unpublished data) and *Aspergillus lentulus* IFM 62627 (Chiba et al., 2021) strains that are infected with partitivirus had been isolated in a previous study. These strains were also treated with 2CMA and 7d2CMA. Of note, colonies free of partitivirus were obtained by treatment with 2CMA at a low rate (<10%), whereas 7d2CMA removed partitiviruses at a high rate.

## Discussion

Artificial treatments are a major strategy for eliminating viruses, and methodologies have been established for limited targets such as human and plant RNA viruses. This study provides the first comprehensive evidence for RNA virus removal in fungi using nucleoside analogs. Overall, 2CMC showed activity against a wider range of mycovirus species compared to ribavirin, 2CMA, and 7d2CMA.

The mycoviruses tested here can be classified into five phyla (Koonin et al., 2020) according to their RdRp sequences: *Pisuviricota* (e.g., *Partitiviridae*, HCV), *Kitrinoviricota* (AfuRV1 can be classified into this phylum), *Lenarviricota* (e.g., *Botourmiaviridae, Narnaviridae, Mitoviridae*), *Negarnaviricota* (AfuNSRV1 can be classified into this phylum), and *Duplornaviricota* (e.g., *Chrysoviridae*). These five phyla ranks cover all non-retro RNA viruses (Kuhlmann et al., 2017), although the classification of *Polymycoviridae* has yet to be established. Our findings revealed that 2CMC enhanced the isolation of virus-free colonies from fungi harboring viruses from the phyla *Pisuviricota*, *Kitrinoviricota*, *Lenarviricota*, and *Duplornaviricota*, although the effect on viruses in *Lenarviricota* was limited. Against viruses in *Negarnaviricota*, 2CMC did not show significant effects. The effect of 2CMC on each virus may be predictable based on its phylum, since the RdRp structure can affect the response to nucleoside analogs. Interestingly, although removing viruses of the *Lenarviricota* and *Negarnaviricota* by nucleoside analogs was difficult, the viruses were spontaneously removed at a low rate.

Viruses in *Partitiviridae* are in general difficult to remove from fungi. For example, AfuPV was not eliminated from *A. fumigatus* by cycloheximide treatment in a previous report (Bhatti et al., 2011). In addition, spontaneous removal of AlePV1 and AfuPV1 was not observed here (Figure 4). Accordingly, studies reporting the elimination of partitiviruses from fungi are limited, with the exception of Kashif et al., where partitivirus was eliminated from a wood decay fungus using thermal treatment (Kashif et al., 2019). The host species might affect the removal rate of partitiviruses. In the present study, we demonstrated that 7d2CMA worked in at least two different filamentous fungi, *A. lentulus* and *A. fumigatus*. It is advantageous if 7d2CMA can remove partitiviruses from a wide range of host fungi.

In our experiments, we used two *A. fumigatus* strains (IFM 63147 and IFM 63431), which harbor AfuNV2. In the case of AfuNV2 infecting IFM 63147, 2CMC successfully enhanced the removal of the virus (17/66). However, in the case of AfuNV2 infecting IFM 63431, no effect of 2CMC was observed (Figure 2; Supplementary Table S2). Considering the multiple infections of mycoviruses in IFM 63147, the removal of AfuNV2 could be enhanced by the presence of other RNA viruses in the same host.

We also used two *A. fumigatus* strains (IFM 62632 and Af293), which harbor AfuPmV-1. The rates of 2CMA to remove AfuPmV-1 from the *A. fumigatus* strains IFM62632 and Af293 were different (Figures 4, 5). In this case, AfuPmV-1 infecting Af293 was removed with high efficiency compared with AfuPmV-1 infecting IFM 62632 which also harbors other two RNA viruses. Although there is a possibility that the multiple infections of mycoviruses in IFM 62632 could affect the removable rate of AfuPmV-1, we also have another hypothesis. Since AfuPmV-1 infecting Af293 has an additional fifth dsRNA segment, which has not been reported in other AfuPmV-1 (Takahashi-Nakaguchi et al., 2020), the presence of this segment in Af293 might be the cause of this difference.

Although we focused on mycoviruses in this study, the nucleoside analogues might be applicable for the elimination of persistent-type RNA viruses in other host organisms. For example, it is known that Mitoviridae-related viruses in *Neopyropia yezoensis* (Red Macroalgae) are widespread among cultivated species for food production (Mizutani et al., 2022). In addition, the arboreal ant *Camponotus yamaokai* is known to harbor Totiviridae-related virus (Koyama et al., 2015). To reveal the function of these viruses, we need to isolate the virus-free strain, like in the cases of mycovirus research. However, no one successfully eliminated the virus to date. It will be valuable to examine the applicability of the nucleoside analogues to other organisms including other fungal species.

All artificial treatments, including stressful conditions, have the potential to provide selection pressure that can fix genetic mutations in both the host and virus. For example, cycloheximide is an inhibitor of protein synthesis and exerts strong growth inhibition, and thermal treatments induce heat stress. Therefore, extreme care should be taken when examining the effects of viruses on a host’s phenotype. To the best of our knowledge, however, the risk of stress introducing genetic changes to fungal genomes that affect their phenotypes has never been verified. The antiviral drugs tested here were shown to hardly affect fungal growth, suggesting that less selection pressure was applied to fungal hosts compared with other known methods. Considering that culturing without artificial treatments poses similar risks, it is absolutely necessary for understanding the effects of viruses on host cells to use multiple independently obtained virus-free strains and to reintroduce viruses into virus-free strains.

In conclusion, we systematically evaluated antiviral drugs for their ability to remove mycoviruses from fungal strains. Although the effects of nucleoside analogs on fungal genomes or fungal RNA viruses are unknown, these antiviral drugs enable us to generate virus-free fungal strains and conduct virus functional studies in an easy and effective fashion.

## Data availability statement

The original contributions presented in the study are included in the article/Supplementary material; further inquiries can be directed to the corresponding authors.

## Author contributions

SU and DH designed the research. AI, YC, and MK performed experiments. AI, SU, and DH analyzed the data. AI, YC, MK, SU, and DH wrote the manuscript. All authors contributed to the article and approved the submitted version.

## Funding

This study was supported by a grant from the Institute for Fermentation, Osaka, and by JSPS KAKENHI (grant no. 20H05579, 21 K18217, 21 K20567, and 22H04879). All sources of funding received for this research was submitted.

## Conflict of interest

The authors declare that the research was conducted in the absence of any commercial or financial relationships that could be construed as a potential conflict of interest.

## Publisher’s note

All claims expressed in this article are solely those of the authors and do not necessarily represent those of their affiliated organizations, or those of the publisher, the editors and the reviewers. Any product that may be evaluated in this article, or claim that may be made by its manufacturer, is not guaranteed or endorsed by the publisher.

## Footnotes

^1^ http://www.nbrp.jp/

^2^ https://jcm.brc.riken.jp/en/
